# Design of the *Focus on Restaurant Engagement to Strengthen Health* (FRESH) study: leveraging systems science to work with independently-owned restaurants to increase access to and promotion of healthful foods

**DOI:** 10.3389/fpubh.2024.1427792

**Published:** 2025-01-07

**Authors:** Uriyoán Colón-Ramos, Emma C. Lewis, Anna Claire Tucker, Lisa Poirier, Chathurangi H. Pathiravasan, Michelle Estradé, Takeru Igusa, Julia A. Wolfson, Yeeli Mui, Veronica Vélez-Burgess, Audrey E. Thomas, Shuxian Hua, Lawrence J. Cheskin, Antonio J. Trujillo, Ayoyemi T. Oladimeji, Stacey Williamson, Rosalinda Romero, Patricia Sánchez Hernández, Joel Gittelsohn

**Affiliations:** ^1^Department of Global Health, George Washington University Milken Institute of Public Health, Washington, DC, United States; ^2^Department of International Health, Johns Hopkins University Bloomberg School of Public Health, Baltimore, MD, United States; ^3^Department of Biostatistics, Johns Hopkins University Bloomberg School of Public Health, Baltimore, MD, United States; ^4^Department of Civil and Systems Engineering, Johns Hopkins University Whiting School of Engineering, Baltimore, MD, United States; ^5^Department of Health Education and Health Communication, Johns Hopkins University Bloomberg School of Public Health, Baltimore, MD, United States; ^6^Department of Nutrition and Food Studies, George Mason University, Fairfax, VA, United States; ^7^Department of Medicine, Johns Hopkins University School of Medicine, Baltimore, MD, United States; ^8^Columbian College of Arts and Sciences, George Washington University, Washington, DC, United States

**Keywords:** systems science, restaurants, dietary quality, food disparities, group model building, formative research, food systems, healthy eating index

## Abstract

**Background:**

High dietary quality can protect against diet-related chronic diseases. In the United States, racial and ethnic minorities and those with lower incomes consistently exhibit lower dietary quality. Independently-owned restaurants are a common prepared food source in minority low-income communities, but there are significant knowledge gaps on how to work with these restaurants to offer healthy food, due to underlying and dynamic complexities associated with providing healthy food options.

**Methods:**

The *Focus on Restaurant Engagement to Strengthen Health* (FRESH) study addresses this complex problem by leveraging systems science approaches to work with independently-owned restaurants. FRESH has two interrelated objectives: (1) to test impact on regular customer dietary quality via a multisite cluster randomized controlled trial in two low-income urban areas (Baltimore and the Washington DC metropolitan area), and (2) to use systems science approaches to develop, parameterize, and calibrate a simulation model. The intervention is theory-and practice-based, comprising three phases: restaurant engagement, low-sugar beverages and healthy meals. The FRESH intervention will be implemented for 12 months in a total of 24 intervention and 24 comparison restaurants. The study is powered to detect a 5-point change in the Healthy Eating Index (HEI) score of regular customers, which would signify a meaningful shift toward healthier eating patterns.

**Discussion:**

The FRESH study will test a novel, multilevel, multisite intervention that aims to improve access to healthier prepared food options among small, independently-owned restaurants located in under-resourced settings. The design of the FRESH intervention and its evaluation are described, as well as plans for the development of a system dynamics simulation model for policymakers and other stakeholders to virtually test future restaurant-based interventions.

**Clinical trial registration:**

https://clinicaltrials.gov, identifier, NCT05869149.

## Background

High dietary quality is a known protective factor against diet-related chronic diseases and all-cause mortality. For example, people with the highest dietary quality exhibit a 23% decreased risk of all-cause mortality compared to those with the lowest dietary quality (1). In the United States, non-Hispanic Black, Latinx, and other populations marginalized by inadequate policies bear an inequitable burden of chronic disease morbidity and mortality related to diet quality (2–4). Previous work to improve dietary quality sustainably has mainly focused on increasing access to healthful foods in retail store settings (5, 6). However, Americans spend about half of their food budget eating out (7, 8), representing at least a 15-fold inflation-adjusted increase since 1970 (9). This trend has contributed an additional 570 kcals to average daily energy intake, owing to both the increased frequency of eating out and larger portion sizes (10). Of the food consumed away from home, 76.8% is purchased at restaurants and fast-food establishments (11). Low-income and minority communities in urban settings are highly exposed to small, independently-owned prepared food sources in particular, most of which predominantly offer energy-dense, nutrient-poor options with few healthier options (12–14). These population groups spend a larger percentage of their food budget at these establishments (12), which are also not subject to policy efforts, such as menu-labeling (15). Working with independently-owned restaurants offers a unique venue to reach customers in low-income minority urban neighborhoods who may benefit most from improved dietary quality. Despite their prevalence in these under-resourced communities, little effort has been directed toward enhancing dietary quality in these establishments (11).

Improving access to and promotion of healthful foods in restaurants presents an inherently multilevel and potentially contradictory problem. Engagement in such efforts is affected by owners’ perceptions of customer demand for these foods, concerns with impact on revenues (e.g., ‘will offering healthy foods to my customers negatively affect sales?’), views concerning community health, and food sourcing challenges (16, 17). Our previous work (18–24) has demonstrated that it is feasible to promote existing healthful food options within independently-owned restaurants, improve their food preparation methods (5), and increase knowledge and acceptability of promoted healthful foods among customers (18, 21). However, community-based interventions tend to be resource-intensive and unsustainable if they do not consider the dynamic complexity across multiple levels of the food system.

The *Focus on Restaurant Engagement to Strengthen Health* (FRESH) study leverages systems science approaches to generate solutions to improve the food environment (25). Systems science addresses complex problems by understanding, identifying, and modeling multiple interacting factors—permitting the virtual testing of potential effects on dietary quality outcomes (26, 27), and saving resources that may otherwise be expended in trial and error (28). As an integral part of FRESH, systems science will inform the development of the trial, and resulting data from the trial will be used to parameterize and calibrate a system dynamics model that can predict the effects of similar strategies in other settings.

Therefore, this manuscript presents the protocol for the two inter-related objectives of this novel study: (1) to refine, implement and test—via a multisite randomized, controlled trial—the impact of the FRESH intervention in independently-owned restaurants located in two largely low-income minority urban sites, and (2) to develop, parameterize, and calibrate a simulation model using data generated from the trial.

## Methods and design

**Figure 1:**
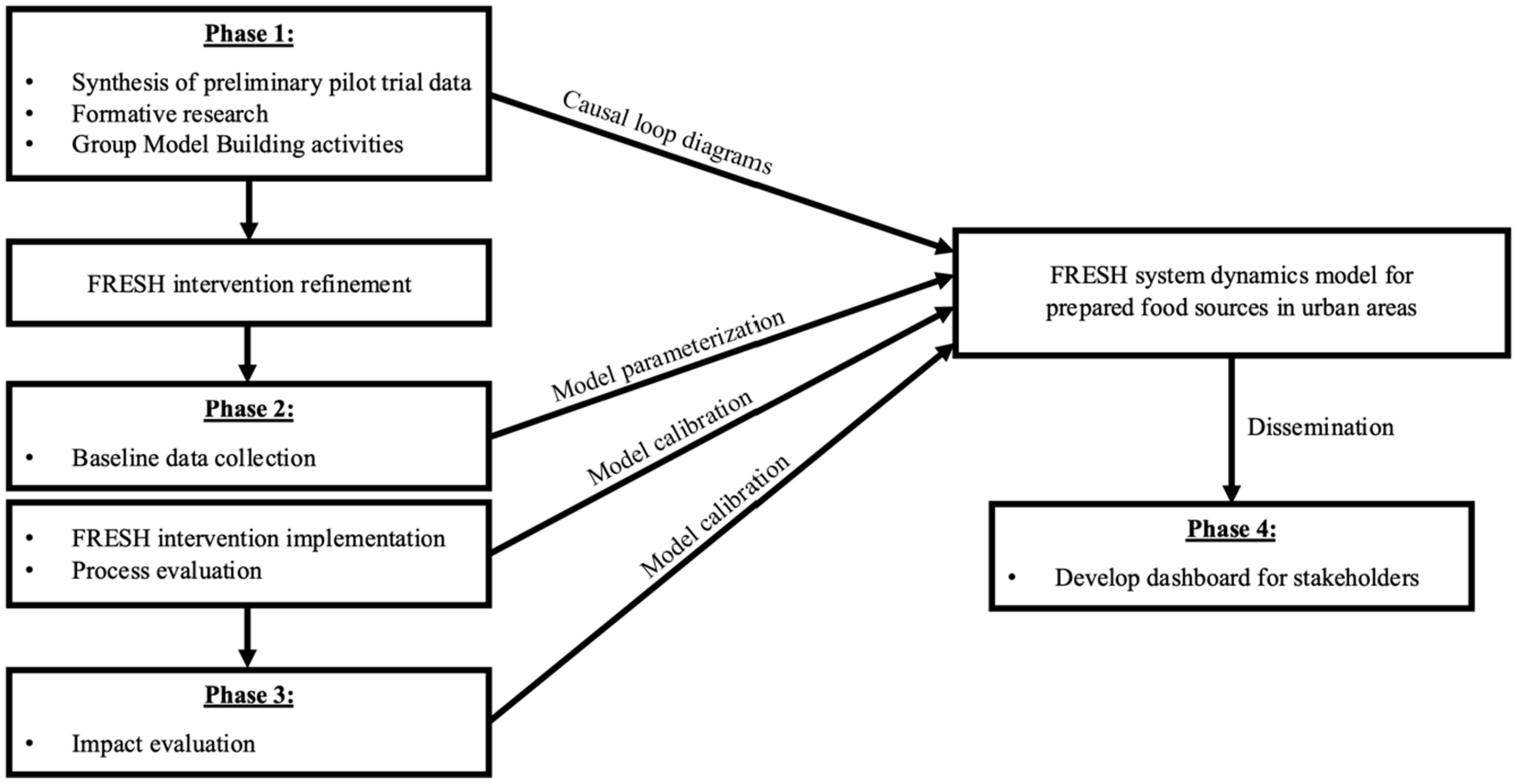
Integration of FRESH intervention study phases with the systems dynamic model.

The FRESH study is innovative in its combination of community-based interventions and systems science—the intervention trial will inform the systems science approaches used (Figure 1). As depicted in the Figure, the two main study objectives will be achieved across four phases. First, comprehensive formative work, including community-based systems science group model building workshops was and will continue to be conducted to refine intervention strategies and develop an initial causal loop diagram. The impact of FRESH will be evaluated via multisite group-randomized controlled trial (RCT) design in two sites—Baltimore, Maryland, and the greater Washington DC metropolitan (DC metro) area—where neighborhoods will be randomly assigned to intervention or comparison conditions. Second, baseline data collected on neighborhoods, restaurants, and their customers will be used to parameterize the initial simulation model and guide the implementation of the intervention. Third, process and impact data from the implementation of FRESH will be used to further calibrate the simulation model. Finally, an interactive, web-based, user-friendly dashboard (29) will be disseminated to policymakers allowing them to apply the system dynamics model to other urban settings.

### Formative and participatory research approaches

The FRESH study leans on academic-community expertise and partnerships with particular experience working with family-style, independent, Latinx-owned restaurants in the DC metro area and in Baltimore (18). This includes leveraging active Community Advisory Boards (CABs) per each site, and using community data collectors and interventionists who are knowledgeable and have deep lived experiences in the communities and neighborhoods in question.

In addition, the FRESH study utilizes extensive formative work to contextualize the trial’s intervention strategies for each site based on qualitative data from: (1) in-depth interviews with restaurant owners/managers and restaurant suppliers (*n* = 31), and (2) structured back-and front-of-house (*n* = 27) and back-of-house observations (*n* = 17).

#### In-depth interviews with restaurant owners/managers

Semi-structured interviews were conducted with the owners/managers of independently-owned restaurants located within predominantly low-income neighborhoods selected at each site. The interview guide was designed to deepen and contextualize our understanding of the restaurants’ and owners’ business history (e.g., length of time in business), business practices (e.g., processes related to suppliers, procurement, delivery, cooking settings, level of training of cooks and willingness to innovate, client-facing staff schedule, restaurant layout), reasons for offering the foods and beverages they do, perceptions of their customers’ reactions toward potential menu changes, and types of promotions they have tested and found to be more or less /successful with their customers.

#### Structured back-of-house observations

Direct observations were carried out in the kitchen or ‘back-of-house’ area (e.g., common areas where foods are prepared, and customers are not allowed). The observation protocol for the back-of-house was designed by the research team for the purpose of this intervention and will capture the number of kitchen staff, cooking equipment, cooking storage, cooking tools, cooking practices as observed (e.g., recipes, types of oil) and availability of specific food groups (e.g., whole grains, fruits without added sugars, vegetables).

#### Structured front-of-house observations

For the ‘front-of-house’, or customer area, observations, we used the NEMS-R (Nutrition Environment Measures Survey-Restaurants) tool (30). This instrument assesses facilitators and barriers to healthy eating, including the restaurants’ promotion of healthful foods that are available on the menu as well as nutrition information, portion options, and other promotional materials.

### Group model-building workshops

**Figure 2:**
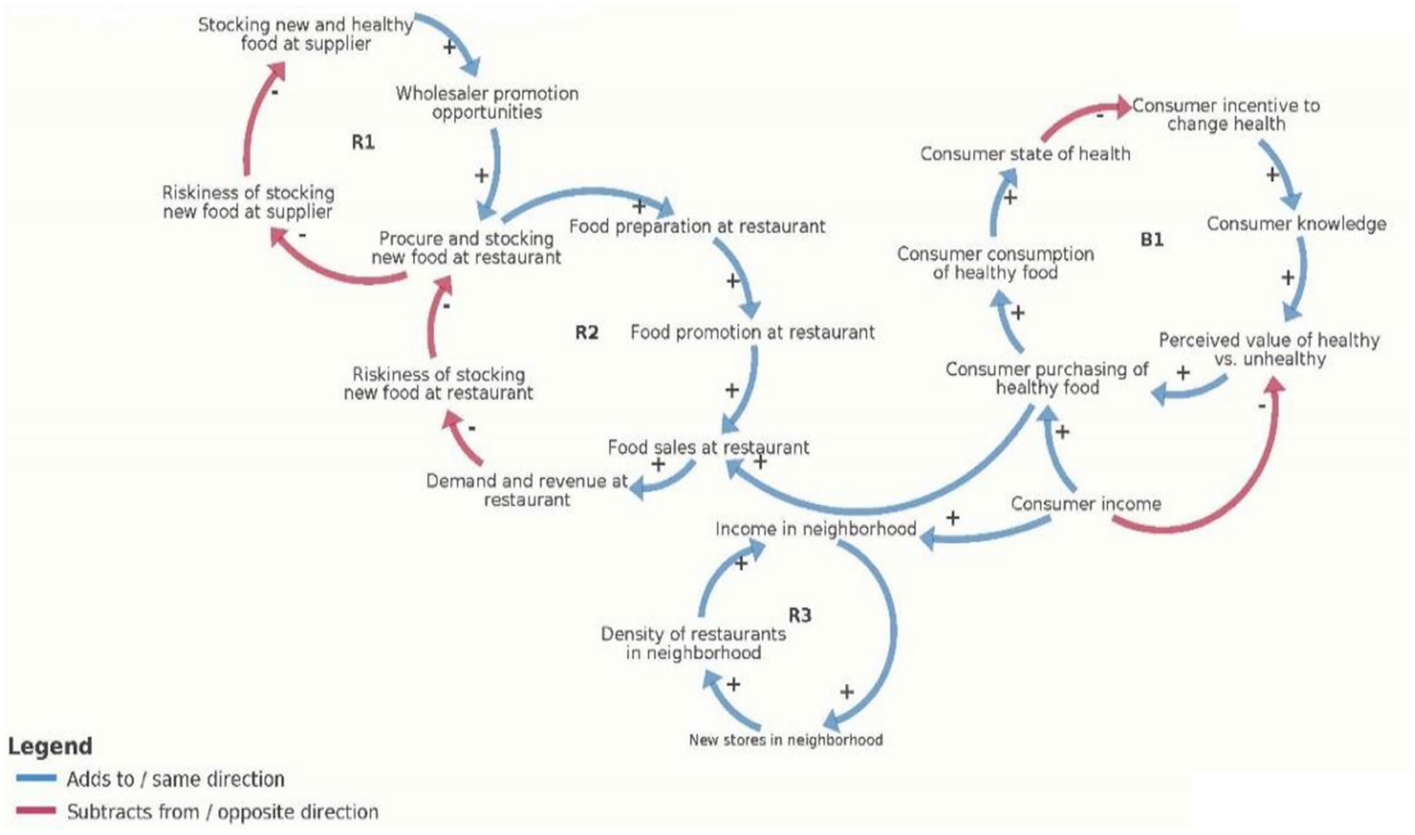
Early causal loop diagram of the restaurant food environment.

Following completion of the formative research, we will conduct a series of group model-building workshops. Group model-building is a participatory approach that elicits participants’ insights into how the restaurants’ systems currently work (including supply procurement, food preparation, service, and interactions with customers), identifying feedback loops to maximize demand, profitability, and healthfulness (31, 32). The first step of the group model building process will be to revise and expand the original causal loop diagram (Figure 2) based on our research team’s experience conducting the formative work—and to produce a revised version (version 2). We will then hold two group model building workshops, one in each research site, where participants will further refine the causal loop diagram (versions 3 and 4). There will be one causal loop diagram for both research sites.

Group model building participants will engage in systems-thinking discussions about key factors that contribute to healthy food availability in restaurants, including the role of food access and procurement, food preparation, and the customer nutrition environment (e.g., the three core components of the FRESH intervention). They will add to and expand the existing CLD based on the formative research, CAB input and the experience of the FRESH team as we begin baseline data collection with restaurants and their owners.

### Site selection, recruitment and baseline data collection

#### Site selection

The study sites are two urban areas: Baltimore, Maryland, and the greater Washington DC metropolitan (DC metro) area. The latter comprises the adjacent urban areas of the District of Columbia, Prince George’s, and Montgomery Counties in Maryland. In total, both sites contain hundreds of restaurants in densely populated areas, including carryout-type restaurants, restaurants with seating/dining areas, and carryouts located in food markets (11).

In Baltimore, neighborhoods (as defined by clustered census tracts) will be selected if they are predominantly (>80%) African American and low-income (with >15% of the population below the poverty level). There are about 1.5 small, prepared food sources per 1,000 residents within city limits, and the majority are independently-owned, carryout-type restaurants (80%) in low-income neighborhoods (11, 33). These restaurants emphasize deep-fried and high-fat foods on their menus, including fried chicken, French fries, pizza, and submarine-style sandwiches.

In the DC metro area, neighborhoods will be selected from clustered census tracts that are both low-income (with >15% of the population below the poverty level) and have a significant Latino population (>35%). These tracts often house smaller neighborhoods that are predominantly (>80%) Latinx, mostly from El Salvador and Guatemala. There are 1.24 restaurants per 1,000 residents in the DC metro area, and many offer primarily Central American foods. These restaurants offer a combination of traditional foods from the owners’ home country, as well as newer dishes, high in saturated fat and sugary beverages.

#### Inclusion and exclusion criteria

Table 1 presents criteria for inclusion in and exclusion from the FRESH trial.

**Table 1:** Inclusion and exclusion criteria for the FRESH trial, by level.

Level	Inclusion	Exclusion
Neighborhood/ clustered census tract	Baltimore study area eligibility criteria: Predominantly (>80%) African American residents Having at least 8 independently-owned restaurants >15% of residents below poverty level DC metro area eligibility criteria: High (>35%) proportion of Latinx residents Having at least 8 independently-owned restaurants >15% of residents below poverty level	Does not meet inclusion criteria
Restaurants	Located within a designated study area (clustered census tract) In good standing with Health Department Inspections Independently-owned In operation for at least 3 years under the same ownership	Franchise of a national, regional, or state chain (e.g., Burger King, KFC, etc.) Very limited hours of operation (open <5 days a week, < 8 h/day) Specialty store (e.g., coffee shop, bakery, donut shop, etc.)
Customers	Adult (18+ years) Regular restaurant customer (buys food > = 1x/week) Live in a household of at least two persons Current resident of study neighborhood	Anticipate moving out of Baltimore or DC metropolitan area in the next 18 months Pregnant

#### Randomization of restaurants

**Figure 3:**
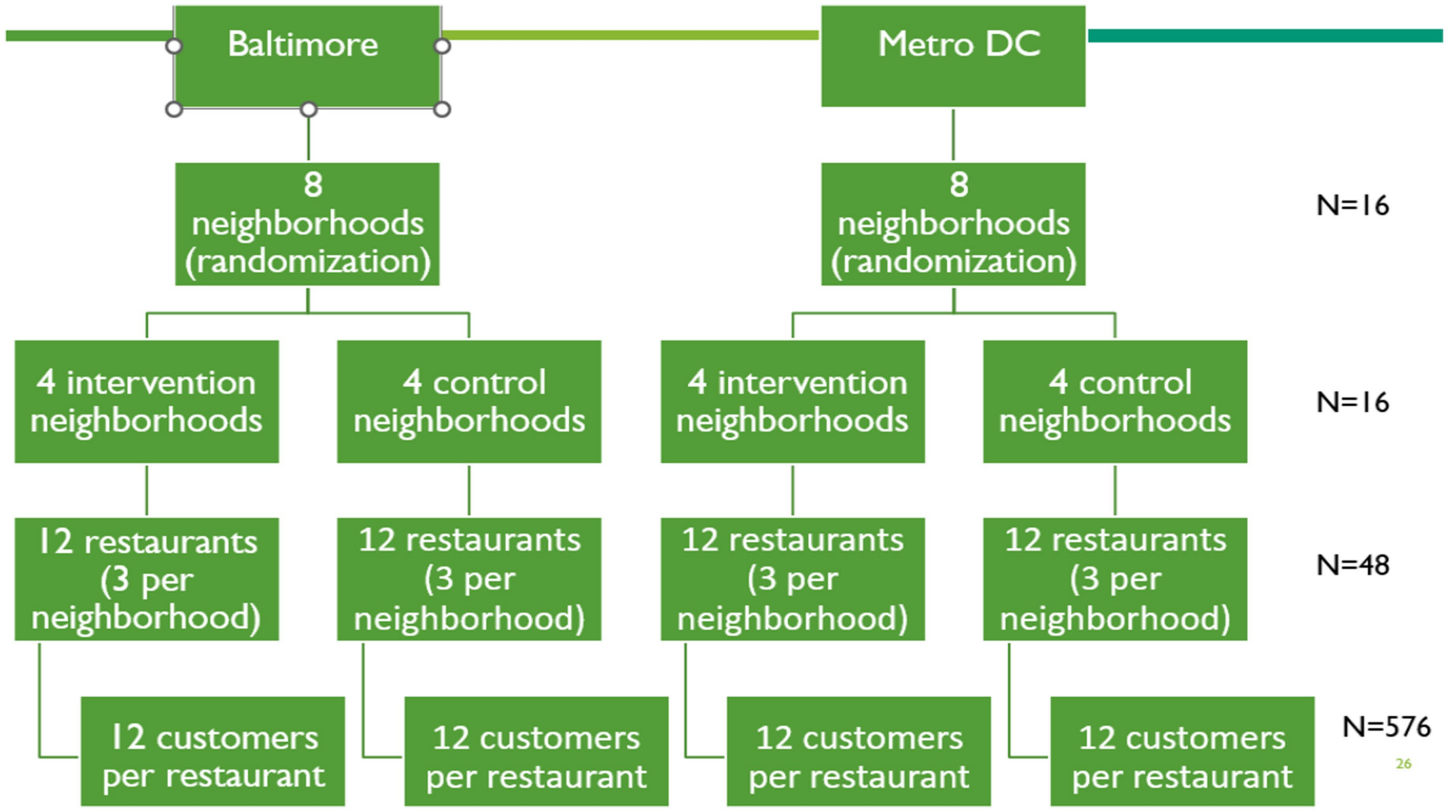
Study design and sample structure of the multi-level FRESH study.

Following completion of the baseline data collection, the 16 census tract clusters will be randomized to intervention or comparison, using a public community-engaged approach we have previously employed in a Native American community setting (34). Half of the restaurants (*n* = 24) will therefore receive the FRESH intervention. At the customer level, half of the customer sample (*n* = 288) will be regular consumers at restaurants receiving the FRESH intervention. The sampling design is presented in Figure 3.

### Development of the FRESH intervention

The FRESH Intervention Working Group will develop the FRESH intervention based on our previous experiences, the results of our formative research, and the input of the FRESH Community Advisory Boards (CABs). Two CABs will be formed, one for Baltimore and one for the DC metro area. CABs will help us stay grounded in community insights by helping us engage with individuals and organizations to maximize the impact of our project, increase project sustainability, and broaden the acceptance of our efforts with communities. Each CAB will be comprised largely of community members, particularly neighborhood association members and restaurant owners, as well as policymakers and food advocacy stakeholders. Members of the CABs will attend CAB meetings to provide feedback on: (1) eligibility of study neighborhoods, (2) identification of restaurants eligible for intervention in neighborhoods, (3) engagement and recruitment strategies, (4) design of data collection tools, (5) design of intervention strategies and materials, (6) how to manage implementation challenges, (7) the group model-building and simulation modeling components, and (8) how best to disseminate and support community initiatives that help improve project sustainability. CAB meetings will be ongoing for the duration of the project. CAB members will determine the frequency of meeting times based on the need to share study milestones and challenges, and when the study team would most benefit from CAB feedback. FRESH CAB members receive a $100 honorarium for their time sharing their input and insights.

### The FRESH intervention

The FRESH intervention will be delivered by three to four trained interventionists over a 12-month period in three phases, each lasting approximately 4 months (Table 2): Engagement, Lower sugar beverages and Healthier meals. Each phase will include activities at the restaurant, consumer and supplier levels. Interventionists will share lived experiences similar to that of individuals working in the independently-owned restaurant businesses (e.g., having owned a restaurant before; working in sales; having a food/nutrition background or interest in restaurants) to prioritize the interventionists’ ability to build rapport with restaurant owners, which will be crucial to obtaining restaurant owner’s buy-in and follow-through on the intervention.

**Table 2:** Overview of FRESH intervention strategies.

Phase of FRESH	Restaurant-level	Consumer-level	Supplier-level
Engagement	Menu changes discussion; preparation of new menu formats (menu board, laminated, QR code, etc.)	Digital marketing selection (social media platform, influencer engagement, website, online delivery, etc.) Digital media to promote restaurant in general Select print media (e.g., table tents)	Initial subsidies to engage restaurants provided in the form of credit at supplier(s) used
Reduced/no sugar beverages	Offer 3 new low/no sugar beverages	Digital media to promote new beverages Print media to promote new beverages	Supplier credit to support costs of offering healthier beverages
Healthy meals	Offer 3 new healthy meals	Social media to promote new meals Print media to promote new meals	Supplier credit to support costs of offering healthier meals

#### Phase 1 (engagement)

This phase is intended to build rapport and trust between restaurant owners, their workers and FRESH team members (FRESH Partners). In a series of planning meetings, FRESH interventionists will meet with each restaurant owner and collectively decide on the best strategies for implementing the FRESH intervention that will be uniquely suited to that restaurant. Discussions will center around identifying three new low-sugar beverages, and three new healthier meals to be offered by the restaurant. These new items will be in addition to any existing healthy choices. A team of Registered Dietitian Nutritionists (RDNs) and graduate-level dietetic students will complete a menu analysis for each restaurant. Menu analysts will work closely with interventionists to identify potential strategies for developing healthier menu items tailored to each restaurant. Specific strategies will be selected on a case-by-case basis by the FRESH Partners and may include collaboratively introducing new recipes or replacing, reducing, or rewriting existing recipes and adjusting cooking techniques toward healthier preparation methods (e.g., baking instead of deep frying). Changes to portion size will also be considered. Based on our formative research, restaurants already have experience using healthier preparation methods, even if these methods are not used for most menu items. Therefore, it was determined that providing restaurant staff with training on healthful cooking methods was unnecessary for the intervention. At the same time, the FRESH Partners will collectively decide on how to revise the restaurant’s menus to highlight and promote these new items (and other healthier items). This may involve professional production of a new menu board, preparation of laminated menus, and/or creation of menu QR code. Discussions during this phase will also cover selection of appropriate digital and print media to promote the restaurant in general. Possible digital media will include a series of social media posts (e.g., on the FRESH Instagram account, by a local influencer), development of a restaurant website (if they do not have one), or updating of their current website. Finally, a small initial subsidy ($500) will be provided to each restaurant to support the purchase of beverages/meal items to consider for promotion, from their local supplier. All costs will be covered by the FRESH project.

#### Phase 2 (reduced/no sugar beverages)

This phase will emphasize the stocking and promotion of three healthier beverages, that are low in added sugar (<5 g/serving) and priced at or below the cost of comparable beverages. Digital and print media will be used to promote beverages identified in Phase 1 to restaurant customers. Intervention restaurants will receive incentives in the form of supplier credit ($750/restaurant) to help offset the risk of stocking these beverages during the promotional period. This incentive will be received after the beverages have been stocked. Lists of targeted beverages will be provided to suppliers to ensure there are sufficient stocks.

#### Phase 3 (healthy meals)

This phase will emphasize the restaurant offering three new healthier meal options, that optimize dietary quality and taste following the DGA (35). A healthier meal is defined as 1 lean protein; AND 1 non-starchy vegetable; AND 1 vegetable (starchy or non-starchy) OR Fruit OR whole grain OR legumes. All components must be non-fried. As with beverages, entrees meeting FRESH criteria should be priced at or below the cost of comparable items. Existing menu items may be modified to meet FRESH criteria by simply adding a new side dish to an existing entrée. Specifically, a restaurant might already sell baked chicken that comes with two side dishes. If the restaurant already offers one side dish meeting FRESH criteria (e.g., collard greens), then the restaurant could develop one additional side dish, such as black-eyed peas, and create a menu item consisting of baked chicken, collard greens, and black-eyed peas to meet FRESH criteria. Digital and print media will be used to promote the three new healthy meals identified in Phase 1 to restaurant customers. Intervention restaurants will receive incentives in the form of supplier credit ($1,500/restaurant) to help offset the risk of providing these meals during the promotional period. This incentive will be received after the three new meals have begun to be sold by the restaurant.

In addition to supplier incentives described above, FRESH intervention staff will support restaurant owners/managers in sourcing the appropriate ingredients for proper FRESH implementation and will work directly with restaurant suppliers to allow them to anticipate stocking needs, a strategy we have used successfully in the past (36, 37). Lists of targeted meal ingredients will be provided to suppliers to ensure there are sufficient stocks.

#### Achieving sufficient intensity during implementation

A key challenge will be to ensure that the FRESH intervention is implemented with sufficient intensity to lead to changes in customer dietary quality. Two strategies will be used to ensure this outcome: (1) all participating restaurants will be required to meet specific minimal standards (e.g., the introduction of at least three low-or no-calorie beverages, and at least three healthy meals); and (2) the FRESH interventionists will meet with owners/managers at least once every 1–2 months to review progress and revise strategies as needed.

#### Process evaluation

We will assess reach, dose delivered, and fidelity (38) of implementation of the FRESH intervention (36). Process evaluation forms will be filled out on REDCap (Research Electronic Data Capture) (39, 40) periodically (e.g., weekly and monthly, depending on the phase of intervention implementation) by interventionists during their regular visits to the restaurants via pre-designed checklist/observation forms and activity logs. Data will be used to calculate indicators for fidelity (e.g., the percent of owners who agree or strongly agree with the beverage stocking option, the percent of items actually stocked), reach (e.g., the percent of customers who recall seeing a promotion, the percent of cook staff trained), and dose delivered (e.g., the number of promotions delivered, the number of training sessions delivered). For each FRESH intervention component, we will divide the resulting process data into tertiles according to low, moderate, or high implementation of each component. We will aim to achieve 100% for all process measure indicators while recognizing that high standards will vary somewhat between restaurants and possibly neighborhoods, and this variation will reflect recommendations for the system dynamics model. Process data for each component of FRESH will eventually be used to calibrate the system dynamics model to guide the intensity of the delivery of future FRESH interventions via machine learning that will be able to assess the ultimate impact of the intervention on final outcomes based on implementation intensity (Phase 4).

#### Impact assessment

Our primary study hypothesis is that regular customers of participating restaurants in FRESH intervention neighborhoods will demonstrate at least a five-point increase in dietary quality as assessed by the Healthy Eating Index 2015 (HEI) score, compared to restaurant customers living in FRESH comparison neighborhoods. We will assess this primary outcome in our consumer sample, as well as a variety of additional secondary outcome measures at the consumer, restaurant and supplier levels. All recruitment materials, consent forms and data collection instruments will be in English and Spanish. The language used will depend on the preferences of the respondent.

#### Selection and training of the evaluation team

Each site will staff a measurement and evaluation team composed of three to four members, separate from the intervention team. The evaluation team will be trained over a three to five-day period prior to collecting survey and anthropometric data to achieve acceptable (>0.90) interrater reliability. Data collectors will be blinded to the treatment group to mitigate risk of bias.

#### Recruitment of the restaurant sample

Forty-eight restaurants will be recruited at baseline according to the specified inclusion criteria. The baseline data collection team will approach the owners/managers of eligible restaurants at their business using a recruitment script that describes the intervention and presents the eligibility criteria.

#### Recruitment of the customer evaluation sample

In each sampled restaurant at baseline, all present customers will be approached by the evaluation team as they exit the restaurant, and, if willing, will be screened for eligibility. The evaluation team will set up a separate time to consent potential customer participants and to collect the survey and anthropometric data at a location convenient for the customer as we have done in other studies (20, 41). The evaluation team will continue to recruit and consent participants on a rolling basis until we have complete data from 12 customers for each participating restaurant (*n* = 576). The names and contact information of all consented participants will be recorded in order to follow them prospectively to conduct the same measures at the end of the intervention (12 months prospectively).

#### Potential for restaurant and customer drop out

It is important to recognize the possibility of low engagement and inadequate participation from restaurants owners, staff, and managers, increasing our risk for low recruitment and restaurant drop out as well as customer drop out. We lean on the experience of our team in working with restaurants and intervention trials in the past and we will employ participatory research approaches (e.g., establishing a CAB per site) as well as relying on support and commitment from community organizations, restaurant suppliers, and Departments of Health to support successful engagement of and transparent communication with independently-owned restaurants. In addition, we have allowed for loss of one restaurant in each neighborhood in our power calculation by having a power of greater than 80% if there are two restaurants. Similarly, anticipating customer drop out, we have oversampled our restaurant customer sample by 20% given experiences in previous trials.

#### Instruments and measures

At baseline, we will collect socio-demographic data and indicators of health status for customers (e.g., age, gender, self-reported health history, education level, socioeconomic status, and household composition), and acculturation (DC metro area only). We will also collect characteristics of restaurants at baseline (e.g., ethnicity of owner, cuisine served). See Table 3.

**Table 3:** Overview of FRESH measures.

Tool used	Outcome	Time frame	Brief description
Primary	Customer dietary quality	Baseline, post-treatment	Regular customer diet quality in the last 30 days will be assessed using the ASA24-Hour Dietary Assessment Tool (ASA24). Healthy Eating Index (HEI) 2015 Score and individual component scores will be calculated from the ASA24 according to the HEI-2015 guidelines to assess changes in overall diet quality and changes within each component.
Secondary	Customer psychosocial constructs	Baseline, post-treatment	Psychosocial outcomes will be measured with scales that have been specifically developed and tested by our study teams, including knowledge, self-efficacy, and behavioral intentions around making healthy choices at restaurants. All scales have acceptable reliability and construct alpha’s (0.63–0.88).
Secondary	Customer restaurant food purchasing behaviors	Baseline, post-treatment	A food purchasing score will be calculated based on the frequency of visiting and ordering foods at participating restaurants in the past 7 days. Emphasis will be placed on documenting purchase of FRESH introduced and promoted beverages, side dishes and entrees.
Secondary	Customer socio-demographics	Baseline	We will collect data on customer age, sex, health history, education, socioeconomic status, household composition, and amount of time living in the United States.
Secondary	Customer skin carotenoid levels	Baseline, post-treatment	Regular customer skin carotenoid levels will be measured using the Veggie Meter®. Three readings will be taken at each data collection point, and the average across the three readings will be calculated and used to assess change from baseline to post-treatment, as an indicator of fruit and vegetable intake.
Secondary	Customer anthropometrics: height, weight, blood pressure	Baseline, post-treatment	Client body weight to the nearest 0.1 lb. will be measured using the Tanita-BIA (Model BF679W). Height to the nearest 0.125 inch will be measured with a stadiometer (Seca 213). Three measures will be made, and the closest two measures averaged. Blood pressure will be measured using the iHealth upper arm cuff (UPC 856362005005).
Secondary	Household food security and food assistance participation	Baseline, post-treatment	We will collect household food security using the 18 question USDA scale, and participation by household members in federal food assistance programs (WIC, SNAP, etc.).
Secondary	Customer exposure to FRESH intervention	Post-treatment	During post-intervention, we will assess exposure by using a standardized form, showing respondents representative materials from each component/phase of the intervention (e.g., posters, flyers, menus) and ask if he/she has seen/heard the material or participated in the activity (e.g., taste test), # times visited participating restaurants, etc. These measures will be recorded in the customer exposure questionnaire. See Supplementary Appendix 2.
Secondary	Restaurant characteristics	Baseline	Restaurant hours, average number of customers, cuisine served, primary spoken language of customers and staff, menu type, beverage availability, age of restaurant, cooking methods used, social media information, number of suppliers, frequency of food procurement/week.
Secondary	Restaurant sales impact	Baseline, post-treatment	We will collect the sales of promoted FRESH foods and beverages of the last week via recall, 9 times during the intervention.

#### Customer-level impact

The primary outcome of dietary quality will be assessed via reported dietary intake from the past 24 h using the ASA24-Hour Dietary Assessment Tool (ASA24). Customers will complete the ASA24 twice at pre-intervention, two weeks apart and this protocol will occur again at post-intervention. The overall HEI score will be calculated according to the methods outlined by the National Cancer Institute (42). All impact measures will be assessed pre-and post-intervention to calculate a change score.

Secondary outcomes at the customer level will be assessed by the Veggie Meter®. This instrument noninvasively measures carotenoid status using a laser for blue light excitation of carotenoids in the skin (43) and has proven validity (44–46). Anthropometric measures will include body weight to the nearest 0.1 kg measured using the Tanita DC-13C scale and height measured to the nearest 1 mm using a stadiometer. Percent body fat will be measured using bioelectrical impedance (Tanita-BIA Model BF679W). Psychosocial constructs will focus on how to make healthy food choices in restaurant settings. *Self-efficacy* will assess the confidence that the respondent feels to perform restaurant-related healthy behaviors. *Intentions* will assess respondents’ intention to perform specified restaurant-related healthy behaviors. Customer Exposure to components of the intervention (dose received) will vary among respondents based on measures such as their use of community media and how frequently they eat at participating restaurants. At post-intervention, we will assess exposure using a standardized form and show representative materials from each component/phase of the intervention and then ask respondents if he/she has seen/heard the material or participated in the activity (47).

#### Restaurant owner/manager impact instruments

General Restaurant Information including restaurant hours, social media usage, age of the restaurant, and staffing information will be collected via short questionnaire from the restaurant owners. The Restaurant environment—Observations of food service style, menu format, as well as menu labeling, amongst other items, will be collected at baseline and post-intervention. Restaurant owners’ psychosocial impact will be measured through self-efficacy and intention questions related to food and carryout operations.

#### Restaurant tracking form

Collected nine times throughout the intervention, restaurant owners will be asked to recall the restaurant ordering of foods that are culturally-relevant and most likely to be ordered as part of the FRESH-approved meals from the last 7 days. Data collectors will ask restaurant owners to recall the restaurant sales of the promoted foods and beverages sold in the last week. They will also report on the number still in stock and the amount of each item that was wasted, as well as perceived customer demand for promoted items.

### Sample size and power

Our power calculations are based on the primary hypothesis, which involves testing the change in mean Healthy Eating Index (HEI) of customers between study groups. Our chosen effect measure is the standardized mean effect of the intervention, representing the difference in mean change in HEI score between the intervention and comparison groups. This measure is standardized by the total variability in the outcome, denoted as the standardized main effect (ES).


$$\begin{array}{l} \mathrm{The standardized main effect}\;\left(ES\right)\\ \;=\frac{\mu_{I}-\mu_{C}}{\sqrt{\tau_{\mathrm{restaurent}}+\tau_{\mathrm{neighborhoods}}+\sigma^{2}}} \end{array}$$

Here, μ_I-μ_C is the mean difference of intervention and comparison groups, respectively. *σ*^2 corresponds to within cluster variation, τ_restaurant corresponds to between restaurant variation within neighborhoods, and τ_neighborhoods corresponds to between neighborhood variation within a site. For our calculations, we assumed a difference in means of 5 points in HEI score would be meaningful, leading to μI − μC = 5. Using data from one of our prior multilevel intervention studies (48), we estimated the standard deviation of the change in HEI outcome to be 9–10 points, giving us σ^^2^ = 9.52^^2^ ≈ 90. We also assumed the variance of change in HEI score between restaurants within a neighborhood, and between neighborhoods within a site, would be small relative to the variance within a restaurant, therefore assuming τ_restaurant = τ_neighborhood≈5_._ Thus, we aimed to be powered to detect an effect size of ES = 0.5. This is equivalent to detecting a medium-sized effect.

Power calculations were conducted using the Optimal Design program (49) and following the documentation for “Multisite cluster randomized trials with treatment at level 3 randomization” section. For all calculations, we assumed a significance level of *α* = 0.05, an effect size of *δ* = 0.5, a total of L = 2 sites (for Baltimore and DC metro area), and an effect size variability of 0, corresponding to fixed effects by site rather than random effects by site.

We varied the number of neighborhoods per site (*K* = 4 to 12), the number of restaurants per neighborhood (*J* = 2,3,4), and the number of customers per restaurant (*n* = 10 to 20). We varied the proportion of variability in the outcome explained by site (Baltimore vs. DC metro area) from 0 to 20% (*B* = 0, 0.1, 0.2). We varied the intra-class correlation coefficients for both the level 2 variable (restaurants) and the level 3 variable (neighborhoods) from 0.01 to 0.1 (ρπ = ρβ = 0.01, 0.05, 0.1). Finally, we assumed a loss to follow-up of 20%. Power is calculated as a function of the number of neighborhoods per site, the number of restaurants per neighborhood, and the number of participating customers per restaurant at initial and follow-up visits. If 8 neighborhoods are randomly assigned to intervention and comparison groups at each site (8 neighborhoods per arm total), the power to detect an effect size a difference of 5 HEI points will exceed 80% if we recruit at least 12 customers per restaurant (*N* = 576) when ICC = 0.05. These calculations account for a potential 20% loss to follow-up.

### Data analysis

The data analysis plan includes a visual review of completed surveys, data entry, data cleaning, exploratory data analysis using sociodemographics, acculturation, baseline health status variables, and multivariable analysis. The statistical plan is to: (1) examine intervention effects via calculating the change variable by subtracting the pretest score/intake from post-test score/intake (exploratory analysis), and then (2) conduct linear mixed-effect models for confirmatory testing of intervention effects across the two study groups.

Exploratory analysis will provide an assessment of the distributions of the outcomes, as well as reveal patterns of missingness and the need for additional data review and quality assurance. If any variable in the final dataset contains more than 5% missing values, we have prepared an imputation plan. To compare the intervention and comparison groups, our primary analysis will use a linear mixed effects model with an individual’s change in HEI score (post-intervention to pre-intervention) as the outcome variable, and including fixed effects for intervention and site, and random effects for restaurant and neighborhood. We will evaluate the effect of the intervention using a hypothesis test on the coefficient of the intervention group variable, which we will interpret as the difference in mean change in HEI score between treatment groups. We will also consider a fixed effect for an intervention-by-site interaction as a secondary analysis to see if the effect of the intervention is different by site (Baltimore vs. DC metro area). Finally, we will consider adjusting these mixed effect models to include customer-level (e.g., age, gender), restaurant-level (e.g., supplier impact questionnaire), and neighborhood-level (e.g., income, restaurant density) covariates.

#### Economic data analyses

We will compare weekly unit sales before and after the implementation using a difference-in-difference approach between intervention and comparison groups and implement an Interrupted Time Series Analysis to explore if changes in the trajectory of sales after the introduction of FRESH can be attributed to the implementation of the program (50). Additional analyses will include sales of promoted foods and ratio of healthy-to-unhealthy food in intervention restaurants.

### Development, parametrization and calibration of the FRESH system dynamics model

We will develop a system dynamics (SD) simulation model that allows stakeholders to test restaurant intervention strategies and policies virtually. Using engineered systems science modeling methodology, the model will integrate the causal loop diagram developed during the group model building activities and parametrize it with FRESH study baseline data on each site, neighborhood, restaurant, and regular customer characteristics. Finally, it will be calibrated using process and impact data from the FRESH intervention RCT. The systems science team will develop a dynamic web-based dashboard for users to interface with the model, to test different intervention strategies and policies, and to assess expected and unexpected effects on customer dietary quality and health, and on restaurant offerings and sales.

#### Causal loop diagram

The initial version of the SD model will be based on the final causal loop diagram developed during the group model-building sessions. This diagram captures the relationships between various customer factors (flows), intervention components, and feedback loops (reverse flows) identified during the sessions. It provides an intuitive visual representation of how these factors interact within the system, including the flows and reverse flows between them.

#### Parameterization of the initial SD model

Publicly available data on parameters at the site and neighborhood levels will be used to parameterize an initial version of the SD model (Table 4). In addition, we will use baseline data from our study to set default values in terms of restaurant and customer characteristics. These parameters will eventually serve to “fit” the intervention to different urban minority sites and simulate the resulting effectiveness of the intervention.

**Table 4:** Potential parameters and calibration factors to be included in the initial SD model.

Levels	Key parameters based on previous work	Data sources
Site (city/county)	Policy requirements to promote healthy eating in restaurants (e.g., menu labeling, healthy beverage in kids’ menus) Frequency of Sanitation/Health Department restaurant inspections	Publicly available
Neighborhood	Density of restaurants Types of restaurants (carryout, sit-down, etc. prepared foods at supermarkets)	Publicly available Census
	Population (age, gender, other sociodemographic such as income level)	

Levels	Example characteristics to calibrate SD model	Data sources (FRESH)
Restaurant	Characteristics (# tables, staff language spoken, # staff, # customers/day, # items in menu, NEMS-R) Food access: # suppliers, frequency procurement, % perishable foods procured, % nonperishable procured Food prep: # equipment in kitchen, # cooks/day	Restaurant impact questionnaire Modified NEMS-R
Customer	Dietary quality (HEI) Prevalence of diet-related chronic diseases, such as cancer Body Mass Index; skin carotenoid levels	Customer impact questionnaire Health status baseline Block FFQ

#### Developing equations to represent stocks and flow relationships

The relationships between customer factors, intervention components, and feedback loops identified in the causal loop diagram will be translated into equations using Ordinary Differential Equations (ODEs) and rational equations. These equations will quantify the flows and accumulated stocks resulting from the FRESH intervention. Flows represent the movement of resources or quantities between different components of the system, such as the quantity of foods stocked, prepared, sold, and consumed. Accumulated stocks represent the capacity or level of certain components, such as the number of healthy foods in storage. By mathematically representing these flows and stocks, the model will provide a quantitative understanding of how the intervention components interact and influence customer behaviors, restaurant operations, and health outcomes over time.

#### Calibrating the SD model

The first round of calibration of the model will occur as we collect data from FRESH process evaluation to reflect the implementation variability that may occur between and within sites. Approximate Bayesian Computation and partial model validation will be used to calibrate and validate the SD model. Face validation will also be performed by demonstrating the model to community representatives and obtaining feedback on the model outputs.

As the data are being collected and cleaned, process indicators will be inputted into the SD model to determine the optimal intensity of future implementations of this intervention based on the following assumptions: (1) with increasing intensity of implementation, consumers will improve their healthy food choices, (2) there is a limit (threshold) after which increasing intensity will not yield substantial improvements in desired effects, and (3) implementing multiple intervention components will reinforce strategies and messages, leading to greater change. The intervention’s intensity-related variables will be calculated as outputs and will be used as references for initial recommendations.

The second round of calibration will use impact findings from the FRESH trial. By comparing initial model predictions with the actual FRESH trial outcomes, the systems science team will improve the model using machine learning techniques in which parameters are estimated (reflecting potential variability in site characteristics) within each component and updated iteratively until convergence is achieved (29). Given the expected multiple flows of the model associated with the different pathways of influence used by the FRESH intervention, we will employ a partial model validation approach to calibrate the system dynamics equations (29). Vensim software will be used to code the computational system dynamics models, including the underlying differential equations.

#### Sharing of results via a user-friendly interactive web-based dashboard

**Figure 4:**
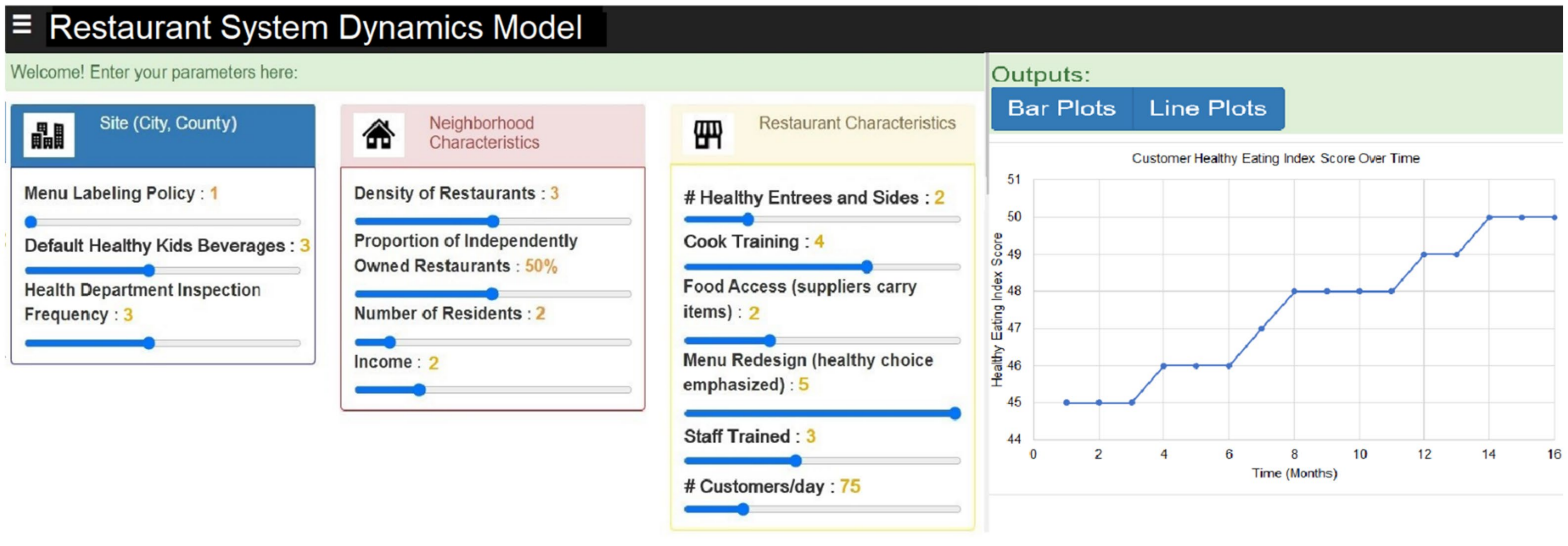
Draft FRESH system dynamics model dashboard and sample output.

Once calibrated, the SD model will be coded using JavaScript and incorporated into a web-based dashboard. The systems science team used a similar approach in developing the web app for the Staple Foods Ordinance (29). In addition to JavaScript, the team will use NextJS and Tailwind (front-end stacks) to build an interactive web-based dashboard to display the simulation results as well as let users input their site parameters, change implementation intensities, and see the eventual effect on HEI and other outcomes of interest which in turn can be manipulated by the users to understand how specific FRESH intervention components would need to be implemented, under which intensity and site parameters, to achieve the specific level of the chosen outcome (e.g., “*how often will a potential customer need to eat at an intervention restaurant in order to see an impact on their overall HEI?”*). Figure 4 shows the input area and an example output of the dashboard, where intervention intensities and site/neighborhood characteristics can be adjusted in the simulation to observe changes in outcomes of interest at the restaurant and customer levels and create visualizations of these outcomes (e.g., consumer HEI).

The dashboard will also feature multiple financial performance metrics, including predicted revenue growth from the sales of healthier menu items and cost evaluations that emphasize possible savings from sourcing and preparing healthier food. A future planned Return on Investment (ROI) calculator will permit restaurant proprietors to enter their particular expenses and anticipated sales growth, facilitating a tailored evaluation of the financial advantages linked to health-oriented initiatives.

The categories and variables in the input area will be adjusted based on the outcomes from the group model building sessions and the final causal loop diagram for the SD model. Information from the dashboard, as well as the FRESH intervention project materials, will be made freely available and downloadable from our project website. The final SD model will be shared with suppliers, restaurant owners, and policymakers to obtain reactions and usability feedback and to encourage them to share among other potential users, in addition to its purposeful dissemination to scientific audiences, policymakers and community users.

## Discussion

Working with independently-owned restaurants is a unique venue to reach people in low-income minority urban neighborhoods who may benefit most from improved dietary quality. The FRESH trial will result in two main outputs: (1) a successful model intervention trial designed to engage independently-owned restaurants in minority neighborhoods, and (2) a user-friendly system dynamics simulation model for virtually testing restaurant interventions. Community-based interventions tend to be resource-intensive and unsustainable if they do not consider the dynamic complexity that characterizes public health problems. Systems science represents a methodological approach suitable for addressing complex problems by understanding, developing, and modeling multiple interacting factors, virtually testing potential effects on health outcomes (26, 27), and saving resources that may otherwise be expended in trial and error (28). This model, which will be made available to the public, will simulate the effects of FRESH on a variety of outcomes, providing the opportunity for others to understand the potential impact of such strategies as applied to their community contexts. FRESH is expected to contribute significantly to the currently limited evidence regarding the use of simulation models to test the impact of restaurant interventions (51), and will provide a tool to inform policymaking decisions for improving the food environment (28). To our knowledge, this is the first application of a systems science approach to develop and visualize the effects of implementing various strategies aimed at increasing access to and promoting healthy eating in small urban restaurants. This is both a significant and innovative step forward for public health intervention science.

Our study does have several limitations. We are recruiting 3 restaurants per neighborhood, leading to the possibility of selection bias. In addition, there is the possibility that working in just a few restaurants in each neighborhood may lead to insufficient intervention intensity. We hope to compensate in part for this challenge by selecting customers who are regular customers of that restaurant. An added limitation is the possibility that intervention and comparison customers may purchase food at the comparison and intervention restaurants at other neighborhoods. To address this, we will assess differences in exposure to the intervention by doing a customer assessment post-intervention of dose received and will examine impact of different levels of exposure on study outcomes as we have done in other studies (52).

## Conclusion

We expect that the results and products from this study will support a shift in how we think about restaurant-based food system interventions, especially in predominantly low-income, minority neighborhoods, and how to engage existing establishments going forward. FRESH will be the first (1) multilevel, multisite restaurant intervention trial to inform a system dynamics model—the result of which will simulate the impact of different FRESH strategies on chronic disease preventive dietary behaviors, subsequently yielding large potential cost savings from avoided trial-and-error intervention work, and (2) intervention that combines previously successful components (e.g., food preparation training, food access improvements, consumer environment) and tests them in independently-owned restaurants.

The FRESH intervention is still in development. It will be implemented starting in late 2024. We anticipate that the trial will successfully demonstrate that an intervention in independently-owned, minority-serving restaurants can have a significant impact on neighborhood customers’ dietary quality. We also anticipate that the system dynamics model will prove to be an invaluable tool for policymakers and other stakeholders to use in their planning of interventions and policies to improve current and future prepared food source environments.
